# An Interactive Web-Based Lethal Means Safety Decision Aid for Suicidal Adults (Lock to Live): Pilot Randomized Controlled Trial

**DOI:** 10.2196/16253

**Published:** 2020-01-29

**Authors:** Marian E Betz, Christopher E Knoepke, Scott Simpson, Bonnie J Siry, Ashley Clement, Tamara Saunders, Rachel Johnson, Deborah Azrael, Edwin D Boudreaux, Faris Omeragic, Leah M Adams, Sydney Almond, Elizabeth Juarez-Colunga, Daniel D Matlock

**Affiliations:** 1 Department of Emergency Medicine University of Colorado School of Medicine Aurora, CO United States; 2 Eastern Colorado Geriatric Research Education and Clinical Center Veterans Health Administration Aurora, CO United States; 3 Division of Cardiology University of Colorado School of Medicine Aurora, CO United States; 4 Adult & Child Consortium for Outcomes Research & Delivery Science University of Colorado School of Medicine Aurora, CO United States; 5 Psychiatric Emergency Services Denver Health Medical Center Denver, CO United States; 6 Department of Psychiatry University of Colorado School of Medicine Aurora, CO United States; 7 School of Public Affairs University of Colorado Colorado Springs Colorado Springs, CO United States; 8 Department of Biostatistics & Informatics Colorado School of Public Health Aurora, CO United States; 9 Harvard Injury Control Research Center Harvard School of Public Health Boston, MA United States; 10 Departments of Emergency Medicine, Psychiatry, and Quantitative Health Sciences University of Massachusetts Medical School Worcester, MA United States; 11 University of Colorado Denver Denver, CO United States; 12 Division of Geriatric Medicine, Department of Medicine University of Colorado School of Medicine Aurora, CO United States

**Keywords:** internet, firearm, suicide, medication

## Abstract

**Background:**

Counseling to reduce access to lethal means such as firearms and medications is recommended for suicidal adults but does not routinely occur. We developed the Web-based *Lock to Live* (L2L) decision aid to help suicidal adults and their families choose options for safer home storage.

**Objective:**

This study aimed to test the feasibility and acceptability of L2L among suicidal adults in emergency departments (EDs).

**Methods:**

At 4 EDs, we enrolled participants (English-speaking, community-dwelling, suicidal adults) in a pilot randomized controlled trial. Participants were randomized in a 13:7 ratio to L2L or control (website with general suicide prevention information) groups and received a 1-week follow-up telephone call.

**Results:**

Baseline characteristics were similar between the intervention (n=33) and control (n=16) groups. At baseline, many participants reported having access to firearms (33/49, 67%), medications (46/49, 94%), or both (29/49, 59%). Participants viewed L2L for a median of 6 min (IQR 4-10 min). L2L also had very high acceptability; almost all participants reported that they would recommend it to someone in the same situation, that the options felt realistic, and that L2L was respectful of values about firearms. In an exploratory analysis of this pilot trial, more participants in the L2L group reported reduced firearm access at follow-up, although the differences were not statistically significant.

**Conclusions:**

The L2L decision aid appears feasible and acceptable for use among adults with suicide risk and may be a useful adjunct to lethal means counseling and other suicide prevention interventions. Future large-scale studies are needed to determine the effect on home access to lethal means.

**Trial Registration:**

ClinicalTrials.gov NCT03478501; https://clinicaltrials.gov/ct2/show/NCT03478501

## Introduction

Identification and intervention with adults at risk of suicide are recommended for health care settings, including emergency departments (EDs) and primary care settings [1]. Reducing access to lethal means for those at risk of suicide is an evidence-based suicide prevention strategy [2,3]. As such, lethal means counseling (LMC) by health care providers is recommended by multiple professional organizations and is a goal of the National Strategy for Suicide Prevention [3].

Firearms are a particular focus for LMC because of their high case fatality rate (85-90%, much higher than other methods) and widespread availability [4,5]. Medical [6,7], suicide prevention [8], and firearm organizations [9] have advocated for securing firearms or removing them from the homes of persons at risk of suicide. Medications are a second key focus of LMC, as overdose is the leading cause of nonfatal suicide attempts [10] and death rates because of opioid overdose are increasing. In addition, including medications in LMC may help improve the acceptability of conversations about firearms [11,12].

Previous work suggests that ED clinicians bring up firearm safety with fewer than half of suicidal patients [13,14]. Likely barriers to counseling include inadequate provider training and awareness along with time demands on busy clinicians; clinicians may also be uncomfortable bringing up a sensitive topic, although previous work shows that patients are generally open to respectful discussion [11,15-17]. Yet the ED is a key acute care setting for suicide prevention, as it is typically the location where patients with suicidal ideation or a suspected suicide attempt are referred for evaluation.

To address these constraints, our team developed the Web-based *Lock to Live* (L2L) decision aid [18] for suicidal adults and their families to consider “which options to choose to reduce home access to firearms or medications” (Multimedia Appendix 1). The L2L Web-based decision aid was developed through an iterative process based on qualitative interviews with key stakeholders, including suicide prevention experts, members of the firearm community, survivors of suicide attempts, and loved ones of suicide victims, as described elsewhere [19]. It includes the typical decision aid sections to help an individual understand a decision (eg, identifying personal preferences and exploring options) and make a choice. Specifically, L2L walks an individual through the rationale for reducing home firearm or medication access and then explores preferences (such as cost) and in- and out-of-home storage options. We hypothesized that a self-administered decision aid to engage ED patients in decision making and augment routine counseling could ultimately enhance patient outcomes, provider satisfaction, and ease of implementation and dissemination. An electronic, Web-based format might facilitate implementation by avoiding the need for paper forms in clinical settings and by allowing confidential engagement for patients waiting in clinical settings; a Web-based format could also allow for broader dissemination to other settings (eg, at home or in outpatient settings).

Here, we describe the results of a pilot randomized controlled trial in EDs that aimed to test the feasibility and acceptability of L2L for adults with suicidal ideation or behavior. Results from this pilot trial can inform the implementation of LMC for adults in EDs and testing of L2L in non-ED settings.

## Methods

### Design and Recruitment

This pilot feasibility trial recruited participants from 4 large EDs in Colorado: a tertiary care academic center, an urban safety net hospital, and a regional medical center with 2 EDs in a geographic region with firearm ownership rates that are higher than state averages. All EDs had 24/7 coverage by behavioral health specialists. Study procedures occurred in the area where the patient was receiving clinical care to limit disruption to ED care and maintain safety precautions. As this was a multisite trial involving a vulnerable population, this study was approved through a full board review of the Colorado Multiple Instructional Review Board, and it was monitored by an external data safety and monitoring board. The trial was registered at Clinicaltrials.gov (NCT03478501).

Eligible participants were English-speaking adult (≥18 years) patients identified as having suicide risk who were not in police custody, who were willing and able to complete telephone follow-up at 1 week (eg, had a working telephone), and who reported ≥1 firearm at home. The study was later expanded to patients with any medications at home (see below). Potentially eligible patients were identified by research assistants (RAs) and approached once deemed medically stable and sober by the treating ED team. Other psychiatric complaints or symptoms (eg, hallucinations) did not preclude eligibility screening, although research staff used discretion in approaching agitated or violent patients. The consent process included questions to determine cognitive capacity to consent. The eligibility and consent script guided the RA to establish rapport, discuss the larger goal of the study (improving home safety generally), and explain participation and confidentiality before asking about firearm ownership.

At each site, participants were a priori block randomized preferentially to the intervention group (13:7 ratio) to increase the amount of feedback on L2L. Randomization occurred after consent to minimize enrollment bias. Participants were blinded but the research staff were not. To blind participants, we used mild deception in the informed consent process, such that patients knew the study was examining ways to enhance home safety of suicidal patients but did not know that L2L was the intervention of interest. The clinical staff were unaware of the treatment group.

At enrollment, participants completed a Web-based baseline questionnaire and then viewed either (1) L2L on a Web-enabled tablet computer or (2) the control, also on the tablet, consisting of general suicide prevention information without a focus on firearm or medication storage. All participants then completed a second questionnaire, including indicating their plan for firearm and medication storage and acceptability questions for the intervention group. Participants could choose to receive a paper printout of either their chosen storage options (L2L group) or the control information. Participating patients were contacted by telephone 1 week after the ED visit for a short questionnaire, which included questions about current firearm and medication storage and changes since the ED visit. Participants who did not answer the telephone for the 1-week follow-up phone call were contacted about once a week thereafter. Participants were considered lost to follow-up at 3 months after enrollment. On the basis of the medical records and vital statistics review, we confirmed that no participants (either contacted or lost to follow-up) had died.

On the basis of the initial feedback and low recruitment, and with institutional review board and data safety monitoring board approval, the study team modified L2L and the study protocol partway through the trial to also address medication safety in addition to firearms. Specifically, L2L incorporated a module on reducing access to medications, and patients could be eligible if they reported medications at home (even without firearms); other eligibility criteria and study procedures were unchanged. The medication module was developed following the same methods used for original L2L development, including stakeholder interviews and iterative refinement [19].

### Measurements

Study data were collected and managed using Research Electronic Data Capture (REDCap), a secure Web system. Participants self-completed questionnaires on tablet computers, and RAs entered additional data (eg, time required to complete L2L) afterward. REDCap was also used for baseline medical record abstraction and telephone follow-up questionnaires (administered by RA).

Key measures assessed in the intervention group included feasibility and acceptability. Feasibility was measured via minutes for the patient to complete L2L as measured by research staff, along with completion rate. Acceptability was measured using the Ottawa Acceptability Scale, a scale measuring comprehensibility (eg, length, amount of information, balance in presentation, and overall suitability for decision making) [20].

Although the pilot trial was not powered to measure the efficacy of L2L on decisions or behavior, for exploratory analysis, we measured (1) decision conflict, a fundamental component of decision quality as a precursor to behavior change [21], and (2) behavior change itself. We hypothesized that patients with higher quality decisions (defined as lower decision conflict) after L2L would be more likely to change their home storage to reduce access to lethal means. Decision conflict was measured using the low-literacy version of the Decisional Conflict Scale (DCS), a 10-item scale with high reliability and test-retest correlation previously shown to discriminate between known groups who make or delay decisions [22]. The DCS scale is scored from 0 to 100, with lower scores indicating less decisional conflict. The baseline and follow-up questionnaires also recorded demographics, living situation, home firearms and medications, and suicide ideation or attempts as measured by the baseline and since-last-visit versions of the Columbia-Suicide Severity Rating Scale [23]. For behavior change, we examined changes in firearm or medication storage between enrollment and follow-up, categorized as *moved in safer direction* (eg, using new or more locking devices or moving items out of the home), *no change*, or *moved in less safe direction* (eg, use of fewer locking devices).

### Statistical Analysis

We used descriptive statistics for feasibility, acceptability, and exploratory analyses on DCS and behavior change. For continuous variables, differences in means between control and intervention groups were tested with 2-sample *t* tests with unequal variances. For categorical variables, we used frequencies with percentages, and differences between groups were tested with Fisher exact test.

## Results

### Participant Characteristics

**Figure 1:**
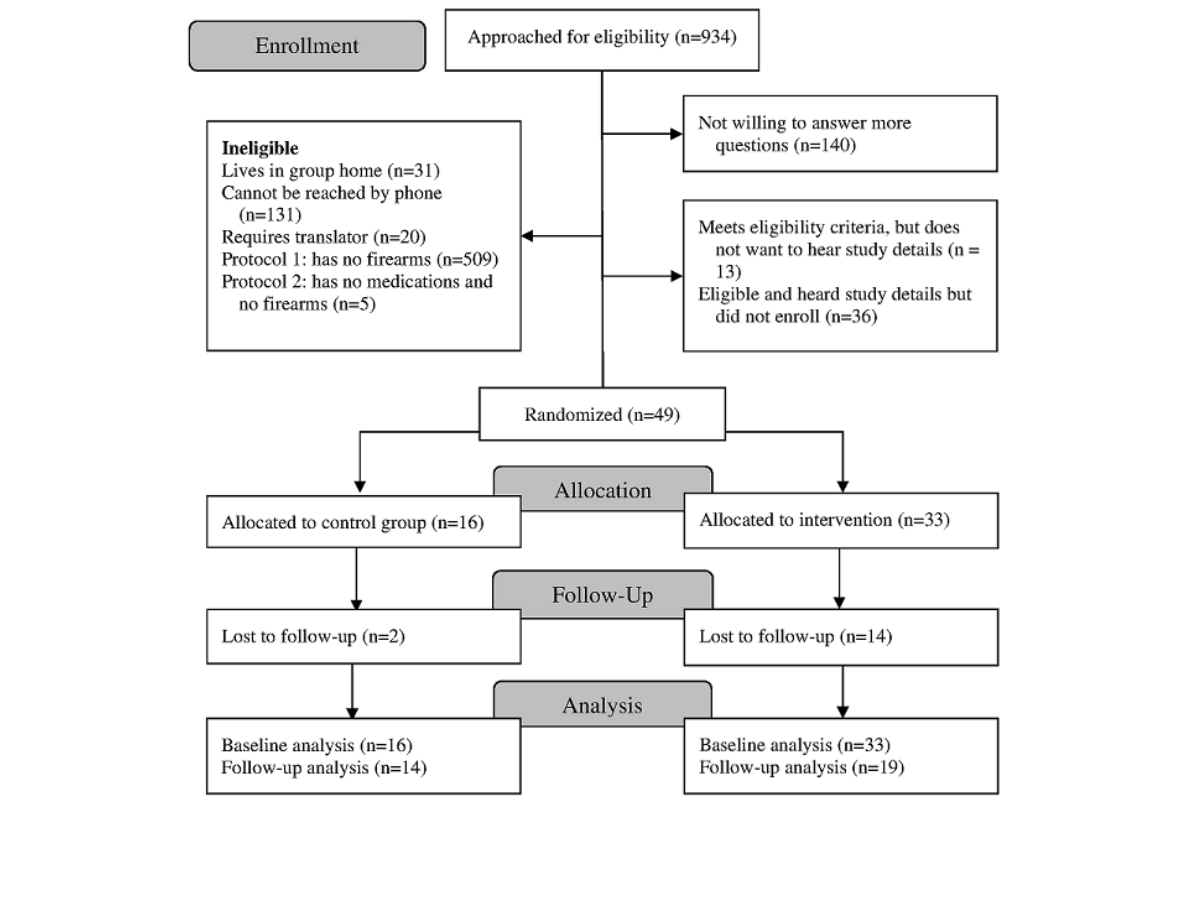
Consolidated Standards of Reporting Trials diagram.

Over 10 months, 49 patients were enrolled, with 33 randomized to the L2L intervention group and 16 to the control group (Figure 1). Overall, patient participant characteristics and study results were similar before and after expanding L2L and eligibility criteria, so results are presented in aggregate. Intervention and control groups were similar on key characteristics and measures (Table 1).

At baseline, 33 of 49 (67%) participants reported having access to firearms at home, 94% (46/49) had medications at home, and 59% (29/49) had both. Of the 33 patients with at least one known firearm at home, 11 (33%) reported 1, 19 (58%) reported more than 1, and 3 (9%) were not sure how many there were. When asked about baseline storage, many of these suicidal adults reported that at least one firearm was unlocked (15/33, 45%), loaded (12/33, 36%), or both unlocked and loaded (8/33, 24%). For the 27 participants with locked firearms, locking devices (eg, cable or trigger locks) were most common (12/27, 44%), followed by firearm safes (9/27, 33%). Of the 47 patients with medications at home, participants reported storing unlocked some or all prescription pain medications (27/47, 57%), over-the-counter pain medications (29/47, 62%), other prescription medications (64%, n=30), and other over-the-counter medications (33/47, 70%). More than half of the 47 enrolled patients with medications at home reported that all were stored unlocked (33/47, 70%).

On the basis of the medical record review, most participants (43/49, 88%) were evaluated by a mental health professional in the ED, and most participants (41/49, 84%) had documentation that at least one provider assessed their access to lethal means. When asked about their ED care, more participants remembered at least one provider talking to them about home firearm access (32/49, 65%) than about medication storage (11/49, 22%).

**Table 1:** Participant characteristics by study group (enrollment).

Participant characteristic^a^		*Lock to Live* (N=33)	Control (N=16)
Age (years), mean (range)		39 (21-68)	38 (21-69)
Male, n (%)		17 (52)	9 (56)
Veteran, n (%)		6 (18)	5 (31)
Currently employed, n (%)		16 (48)	9 (56)
≥1 child at home, n (%)		8 (24)	6 (38)
Currently receiving outpatient mental health care, n (%)		7 (21)	7 (44)
**Race, n (%)**			
	White	23 (70)	10 (63)
	Black	5 (15)	0 (0)
	Other or not documented	5 (15)	6 (38)
	Hispanic ethnicity	5 (15)	4 (25)
**Highest grade completed, n (%)**			
	High school graduate or less	9 (27)	4 (25)
	Vocational or technical school graduate or some college	14 (42)	10 (63)
	College graduate or higher	10 (30)	2 (13)
**Current marital status, n (%)**			
	Never married	15 (45)	4 (25)
	Married	10 (30)	8 (50)
	Widowed, divorced, or other	8 (24)	4 (25)
**Columbia-Suicide Severity Rating Scale, n (%)**			
	Lifetime	12.9 (6)	14.4 (5.7)
	Past week	16.4 (4)	14.8 (5.9)
**Medical record documentation, n (%)**			
	Alcohol abuse	4 (12)	5 (31)
	Alcohol intoxication	6 (18)	5 (31)
	Intentional illegal or prescription drug use	11 (33)	3 (19)
**Past week (including current visit), n (%)**			
	Suicide ideation	31 (94)	13 (81)
	Suicide attempt	12 (36)	6 (38)
	Homicidal ideation	3 (9)	1 (6)
	Interpersonal violence	3 (9)	1 (6)
**Lethal means access, n (%)**			
	Not documented	5 (15)	3 (19)
	No access to lethal means	4 (12)	1 (6)
	Access to lethal means	20 (61)	10 (63)
	Firearms specifically mentioned	18 (90)	10 (100)
**Way to reduce access to lethal means discussed, n (%)**			
	Yes, documented in medical record	9 (27)	9 (56)
	No, documented in medical record	12 (36)	2 (13)
	Not documented in medical record	9 (27)	3 (19)
Evaluated by mental health professional during visit, n (%)		29 (88)	14 (88)
Written safety plan, n (%)		3 (9)	0 (0.0)
**Decision outcomes, mean (SD)**			
	Total DCS^b^ score (out of 100)	12.6 (20)	9.7 (18)
	DCS score <25^c^	29 (88)	13 (81)
**Likelihood of changing storage at home (out of 7), mean (SD)**			
	Firearms	3.5 (2.5)	4.2 (2.4)
	Medications	3.6 (2.3)	4.1 (2.5)

^a^Numbers and percentages may not sum to total because of missing data (not shown if <5%) or questions allowing ≥1 response.

^b^DCS: Decisional Conflict Scale.

^c^Scores <25 previously associated with implementing decisions [22].

### Intervention Feasibility and Acceptability

**Figure 2:**
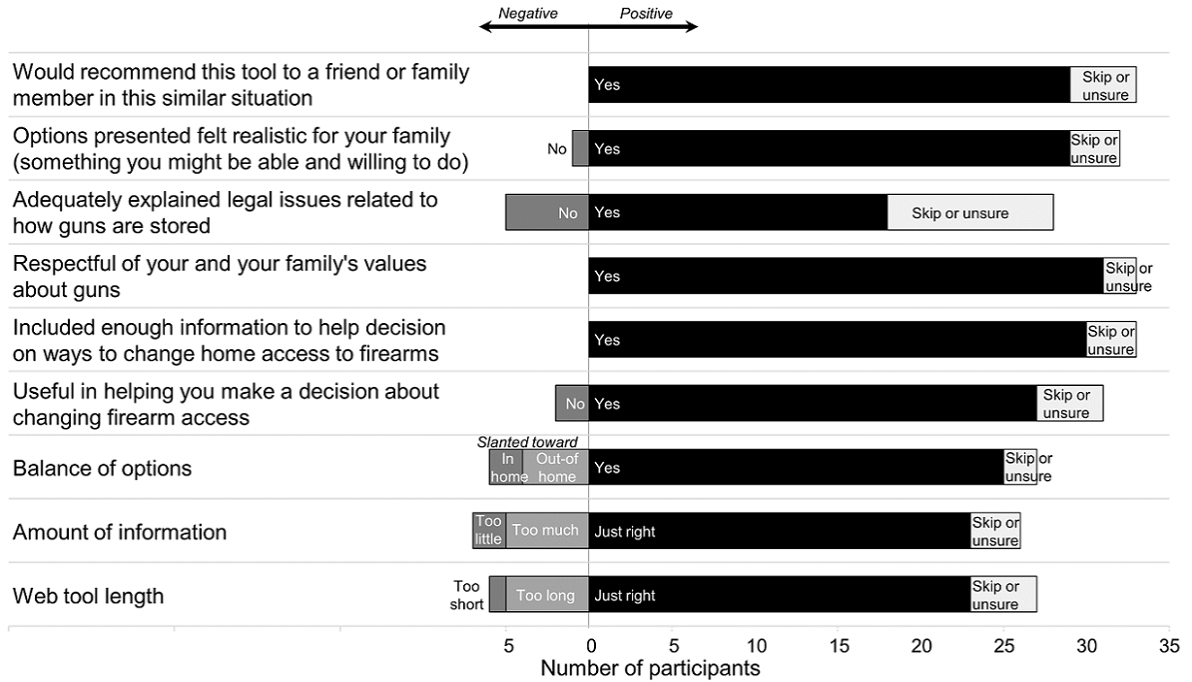
Lock to Live acceptability (n=33).

Feasibility and acceptability of the L2L intervention were excellent. All intervention group participants (n=33) completed L2L, with a median viewing time of 6 min (IQR 4-10 min), and most (24/33, 73%) of the participants wanted a printout of the last page with the final choices and recommendations. Most participants (31/33, 94%) viewed L2L by themselves, without a family or friend present and without a provider. Multimedia Appendix 2 displays selected storage options; participants made 53 selections in L2L, in addition to *friends or family* (which was selected by default but could be unselected; 10 participants unselected it). Figure 2 shows responses to the Ottawa Acceptability Scale. Almost all participants reported that they would recommend the tool to a friend or family member in the same situation, that the options felt realistic, and that L2L was respectful of values about firearms. Areas for improvement included explanation of legal issues, as 15% (5/33) of the participants reported that the tool did not adequately explain legal issues and 30% (10/33) of the participants were unsure or skipped the question. Most of the participants felt that the tool had the right length and had the right amount of information, with a balance in the presentation of options.

In the exploratory analysis, there were no significant differences in decision conflict or planned storage changes between the L2L and control groups. DCS scores were low in both groups (suggesting low decision conflict), and participants gave overall neutral responses when asked about the likelihood of changing either firearm or medication storage (mean 3.8 out of 7). When asked about planned changes to storage, 8 participants overall said that they would use more lockboxes at home or dispose of unneeded medications (8/31, 26% each), with no difference between the L2L and control groups. For firearms, participants across the L2L and control groups most commonly said that they would use more firearm locking devices (14/22, 64% overall) or safes or lockboxes (13/22, 59% overall) at home; the most common out-of-home options were storing firearms with a trusted family member (13/22, 59% overall) or friend (9/22, 41% overall). Fewer participants cited storage with firearm stores or ranges or with law enforcement (3/22, 14% for each) as likely.

Two-thirds (n=33; n=14 control and n=19 intervention) of the participants completed telephone follow-up (Figure 1) at an average of 2.4 weeks (SD 2.2; range 1-9 weeks) after enrollment; among these, 25 participants reported on firearm storage and 6 on medication storage. There were 14 participants who reported having firearms at both baseline and follow-up (including 3 who had moved firearms out of the home). Among these 14, as compared with the control group, more participants in the L2L group had moved in a safer direction (1/7, 14% vs 4/7, 57%), although the difference was not statistically significant. Similarly, as compared with the control group, fewer participants in the L2L group had made no change (5/7, 71% vs 3/7, 43%) or had moved in a less safe direction (1/7, 14% vs 0/7, 0%). These differences were also not statistically significant.

## Discussion

### Principal Findings

In this pilot trial, the acceptability of the L2L decision aid was very high among adults with acute suicide risk. Although the trial was not powered to identify an effect on subsequent home lethal means access, L2L appears feasible for clinical use in that it took a median of 6 min, and there were no major issues accessing the content via a tablet in the ED. Questions remain, however, about how best to implement its routine use in clinical settings for appropriate patients.

Participants overwhelmingly found L2L to be useful, informative, and respectful of their values with regard to firearm ownership. This high acceptability supports further clinical application and evaluation of L2L. Future research should address effects on decision making and subsequent lethal means access, along with methods for implementation and dissemination. It may be that L2L would have the greatest effect when used within a conversation with a provider, after rapport has already been established. In such a role, L2L might support counseling and decision making by helping an individual clarify values and understand logistics that the counselor may not be fully knowledgeable about.

On the other hand, there is evidence that patients may not disclose suicidality [24] or firearm ownership to providers, so upstream, community-based (nonclinical) approaches may also be useful to disseminate messages to those at risk. Indeed, although designed and pilot tested in ED settings, L2L does not include language specific to the ED setting and therefore could be tested in other clinical or community settings. Active studies are testing L2L in outpatient primary care and outpatient mental health settings, but integration into broader public education campaigns also deserves consideration. Such campaigns should incorporate information intended to raise awareness and change beliefs concerning the importance of reducing access to lethal means during times of suicide risk [25]—beliefs previously suggested to be associated with firearm storage behaviors [26,27].

Indeed, our pilot findings raise questions about whether suicidal adults in EDs recognize or believe that lethal means access is an issue in need of a decision or behavior change. Decision conflict scores were relatively low in both groups, with 86% (29/33) of participants overall having scores less than 25. In previous work, low decision conflict scores—indicating low internal conflict about the decision—have been associated with implementing decisions [22]. It may be that L2L would have optimal effects when integrated into care after delivery of LMC by providers, as such counseling might prime the individual for decision making. Pairing with other interventions to overcome obstacles in making storage changes—be they financial, logistical, or emotional—may also be useful. For example, in this pilot study, we did not test the effect of providing locking devices or contact information for nearby out-of-home storage locations, but such tangible add-ons may help motivate change. Previous work suggests that provision of locking devices can boost responses to LMC [28]; similarly, facilitating connection to local storage partners may overcome logistic barriers [29].

Testing the role of family or friends was a challenge in this study. There is real-world variability in how family or friends are involved with lethal means safety counseling; ideally, the person with decision-making control over storage would be involved in the ED, but they may not be present with the patient. The low observed rates of presence and participation by trusted individuals could reflect that suicidal adults may have thought distortions such that they do not want to *burden* family or friends by engaging them in research or being in the ED with them. Low referral of family or friends may also have stemmed from confidentiality concerns or fear of firearm confiscation. Recognizing the importance of engaging trusted others, the L2L tool includes a section where patients are prompted to consider who they would enlist to help implement their storage plan (eg, family members, friends, religious leaders, or fellow Veterans). Our findings, and the questions they raise, highlight the need for strategies to better enlist patients’ caring contacts in both clinical care and research participation.

### Limitations

By their nature, pilot studies do not offer power to examine primary efficacy outcomes, so we designed the trial to provide information about feasibility. We cannot comment on L2L’s effect on suicide-related outcomes, and we did not examine other predictors of these outcomes. The trial itself did not engage ED providers, who would be key partners in the implementation of the intervention into clinical practice; a related mixed method study is underway. Another limitation is blinding, as RAs had to know group assignment to ensure the participant could access L2L. Concerns about confidentiality expressed by stakeholders involved in the design process led to the a priori decision to not collect these data through the tool itself, however [30].

### Conclusions

The line of investigation exemplified by this trial offers the possibility of better facilitating lethal means safety counseling in a patient-centered, acceptable, and feasible way. The Web format and respectful messaging offer a way for medical and mental health providers to augment LMC by providing a simple, patient-centered tool with which to present various safe storage options. For providers who are unfamiliar with options or uncomfortable with these discussions, L2L might increase their willingness to address lethal means access with at-risk patients; further examination of L2L’s effect on provider behavior is warranted. Critically, if L2L is found effective in future work, its Web format offers the potential for rapid and widespread dissemination and integration into suicide prevention efforts as well as more rapid updating as indicated (eg, for new relevant legislation). Future mixed methods research examining tool effectiveness and implementation could help enhance home safety and prevent suicide.

## Appendix

Multimedia Appendix 1 Representative screenshots of Lock to Live.

Multimedia Appendix 2 Firearm storage options chosen by intervention participants after viewing Lock to Live (Multiple responses are allowed. Both family, friend, or neighbor and lock box were selected as defaults. Participants could unselect them if they chose).

Multimedia Appendix 3 CONSORT-EHEALTH checklist (V 1.6.1).

## Abbreviations

DCS: Decisional Conflict Scale
ED: emergency department
L2L: Lock to Live
LMC: lethal means counseling
NIH: National Institutes of Health
RA: research assistant
REDCap: Research Electronic Data Capture
