# Supplementary material for: An Interactive Web-Based Lethal Means Safety Decision Aid for Suicidal Adults (Lock to Live): Pilot Randomized Controlled Trial
Source: J Med Internet Res. 2020 Jan 29;22(1):e16253. doi: 10.2196/16253 (PMC7016618; doi:10.2196/16253)
Supplement: Multimedia Appendix 1 [file jmir_v22i1e16253_app1.pdf]

FAQ TALK TO SOMEONE

Lock to Live

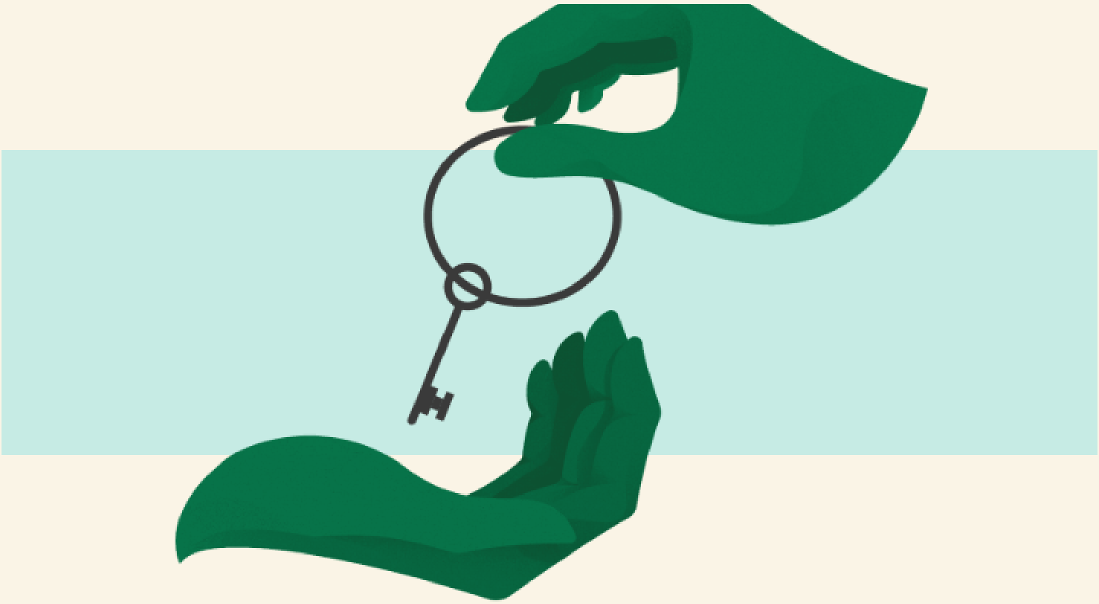

You or someone you know may feel hopeless, down, or alone right now. Many people have gotten through times like this, and you can too.

This tool can help you make decisions about temporarily reducing access to potentially dangerous things, like firearms, medicines, sharp objects, or other household items.

The promise of tomorrow can start with small steps towards safety today.

Representative screen shot. Tool available at https://lock2live.org
© 2018 The Regents of the University of Colorado

FAQ TALK TO SOMEONE

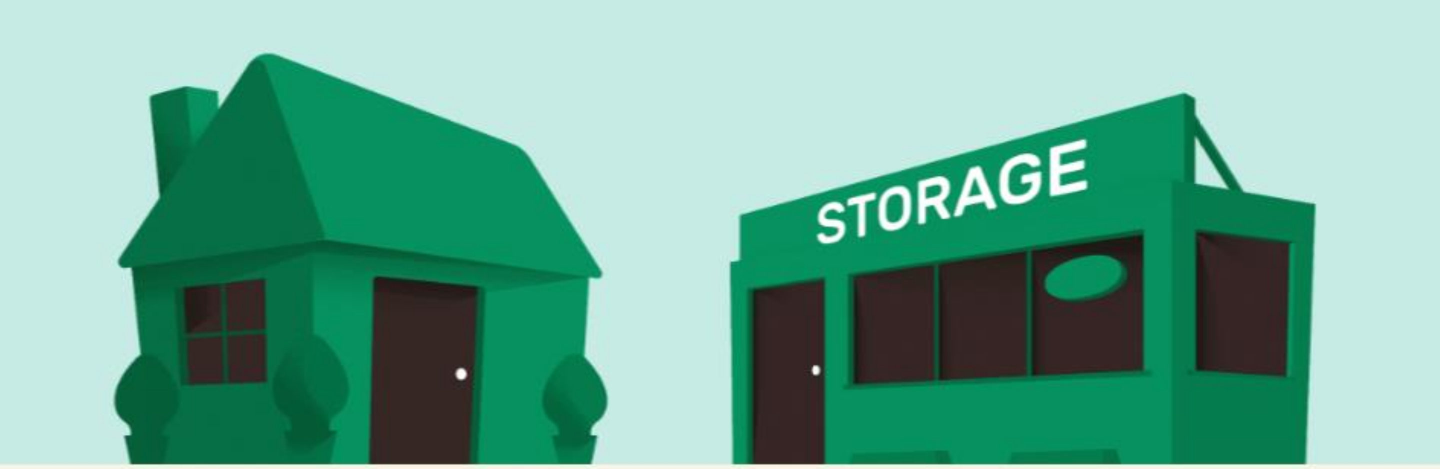

All firearm storage options fall into one of two types, secured either: at home or temporarily away from home.

The safest storage method is the one that will make a firearm hardest to reach during a moment of crisis.

Representative screen shot. Tool available at https://lock2live.org

© 2018 The Regents of the University of Colorado

FAQ TALK TO SOMEONE

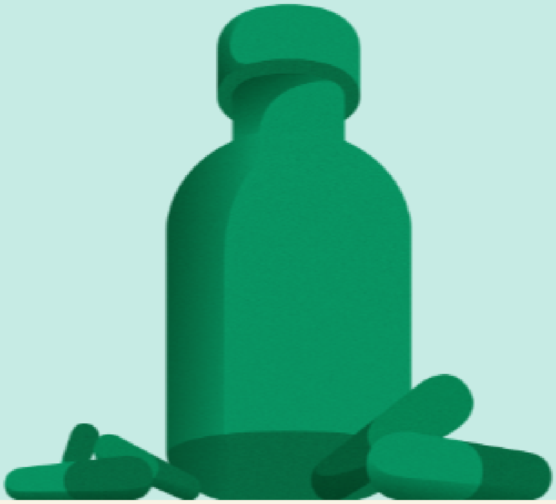

Medications are often an important part of daily life.

Some people need to take medications many times a day, while others may just need them for emergencies. Think about the medications where you live and answer the following questions:

Representative screen shot. Tool available at https://lock2live.org

© 2018 The Regents of the University of Colorado

Let's Talk About Other Hazards

Other household hazards can be hard to lock up and store during difficult times, but it's still a good idea to talk about them. These can include things like:

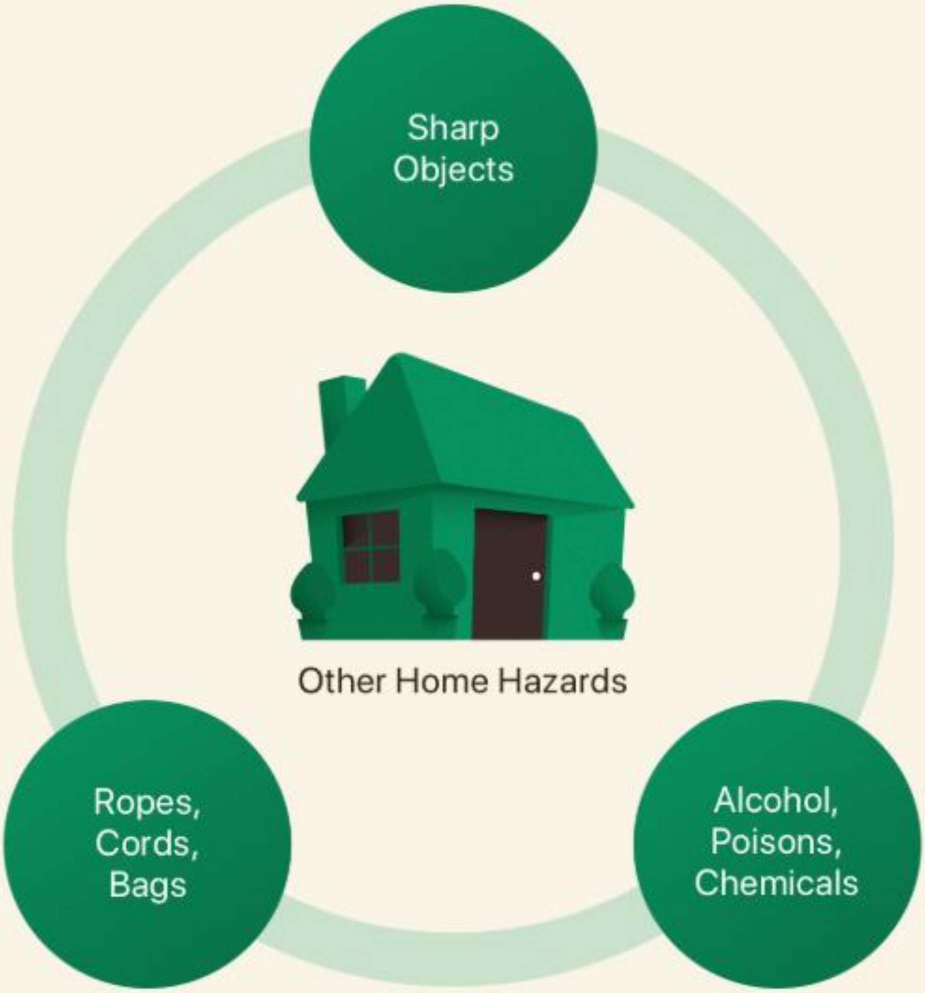

Many of these items are found around the house, used everyday, or can be difficult to keep track of.

Representative screen shot. Tool available at https://lock2live.org

© 2018 The Regents of the University of Colorado
