# Supplementary figures and images for: An Interactive Web-Based Lethal Means Safety Decision Aid for Suicidal Adults (Lock to Live): Pilot Randomized Controlled Trial
Source: J Med Internet Res. 2020 Jan 29;22(1):e16253. doi: 10.2196/16253 (PMC7016618; doi:10.2196/16253)

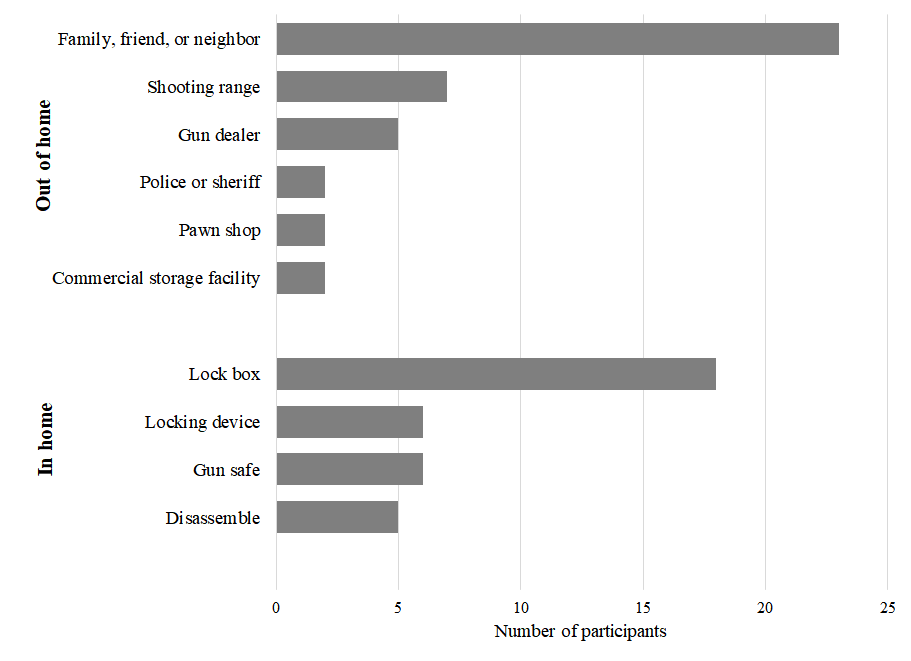

Supplement: Multimedia Appendix 2 [file jmir_v22i1e16253_app2.png]
