# Supplementary material for: An Interactive Web-Based Lethal Means Safety Decision Aid for Suicidal Adults (Lock to Live): Pilot Randomized Controlled Trial
Source: J Med Internet Res. 2020 Jan 29;22(1):e16253. doi: 10.2196/16253 (PMC7016618; doi:10.2196/16253)
Supplement: Multimedia Appendix 3 [file jmir_v22i1e16253_app3.pdf]

CONSORT-EHEALTH Checklist V1.6.2 Report	Manuscript Number	16253

(based on CONSORT-EHEALTH V1.6), available at [http://tinyurl.com/consort-ehealth-v1-6].

Date completed

9/13/2019 13:41:54

by

Marian Betz

“Lock to Live”: A pilot randomized trial of an interactive web-based lethal means safety decision aid for suicidal adults for use in the emergency department

TITLE

1a-i) Identify the mode of delivery in the title

"A pilot randomized trial of an interactive web-based lethal means safety decision aid"

1a-ii) Non-web-based components or important co-interventions in title

"for use in the emergency department"

1a-iii) Primary condition or target group in the title

"for suicidal adults for use in the emergency department"

ABSTRACT

1b-i) Key features/functionalities/components of the intervention and comparator in the METHODS section of the ABSTRACT

"Participants were randomized in a 13:7 ratio to L2L or control (website with general suicide prevention information) and received a one-week follow-up telephone call."

1b-ii) Level of human involvement in the METHODS section of the ABSTRACT

1b-iii) Open vs. closed, web-based (self-assessment) vs. face-to-face assessments in the METHODS section of the ABSTRACT

"At four emergency departments, we enrolled participants"

1b-iv) RESULTS section in abstract must contain use data

"Baseline characteristics were similar between the intervention (n=33) and control (n=16) groups. At baseline, many reported having access to firearms (67%), medications (94%), or both (59%). Participants viewed L2L for a median of 6 minutes (interquartile range: 4-10 minutes). L2L also had very high acceptability; almost all reported they would recommend it to someone in the same situation, that the options felt realistic, and that L2L was respectful of values about firearms. In exploratory analysis in this pilot trial, there were no significant"

1b-v) CONCLUSIONS/DISCUSSION in abstract for negative trials

"The “Lock to Live” decision aid appears feasible and acceptable for use among adults with suicide risk and may be a useful adjunct to lethal means counseling and other suicide prevention interventions. Future large-scale studies are needed to determine effect on home access to lethal means."

INTRODUCTION

2a-i) Problem and the type of system/solution

"To address these constraints, our team developed the web-based “Lock to Live” (L2L) decision aid for suicidal adults and their family to consider “which options to choose to reduce home access to firearms.” A self-administered decision aid to engage ED patients in decision-making and augment routine counseling stands to enhance patient outcomes, provider satisfaction, and ease of implementation and dissemination."

2a-ii) Scientific background, rationale: What is known about the (type of) system

"Prior work suggests that ED clinicians bring up firearm safety with fewer than half of suicidal patients [14,15]. Likely barriers to counseling include inadequate provider training and awareness along with time demands on busy clinicians; clinicians may also be uncomfortable bringing up a sensitive topic, though prior work shows patients are generally open to respectful discussion [12,16-18]."

"An electronic, web-based format facilitates implementation by avoiding the need for paper forms in clinical settings and by allowing confidential engagement for patients waiting in clinical settings; a web-format also allows for broader dissemination to other settings (e.g., at home or in outpatient settings). "

Does your paper address CONSORT subitem 2b?

"Here we describe the results of a pilot randomized controlled trial in EDs that aimed to test the feasibility and acceptability of L2L for adults with suicidal ideation or behavior. Results from this pilot trial can inform implementation of LMC for adults in EDs and testing of L2L in non-ED settings."

METHODS

3a) CONSORT: Description of trial design (such as parallel, factorial) including allocation ratio

"This pilot feasibility trial recruited participants from three large EDs in Colorado: "
"At each site, participants were a priori randomized preferentially to the intervention group (13:7 ratio) to increase the amount of feedback on L2L"

3b) CONSORT: Important changes to methods after trial commencement (such as eligibility criteria), with reasons

"Based on initial feedback and low recruitment, and with IRB and DSMB approval, the study team modified L2L and the study protocol partway through the trial to also address medication safety in addition to firearms. Specifically, L2L incorporated a module on reducing access to medications and patients could be eligible if they reported medications at home (even without firearms); other eligibility criteria and study procedures were unchanged. "

3b-i) Bug fixes, Downtimes, Content Changes

There were none

4a) CONSORT: Eligibility criteria for participants

"Eligible participants were English-speaking adult (≥18 years) patients identified as having suicide risk who were not in police custody, able to complete telephone follow-up at one week, and who reported ≥1 firearm at home."

4a-i) Computer / Internet literacy

4a-ii) Open vs. closed, web-based vs. face-to-face assessments:

"Potentially eligible patients were identified by research assistants (RAs) and approached once deemed medically stable and sober by the treating ED team. Other psychiatric complaints or symptoms (e.g., hallucinations) did not preclude eligibility screening, though research staff used discretion in approaching agitated or violent patients. The consent process included questions to determine cognitive capacity to consent. The eligibility and consent script guided the RA to establish rapport, discuss the larger goal of the study (improving home safety generally), and explain participation and confidentiality before asking about firearm ownership."

4a-iii) Information giving during recruitment

No information given during recruitment

4b) CONSORT: Settings and locations where the data were collected

"This pilot feasibility trial recruited participants from three large EDs in Colorado: a tertiary care academic center, an urban safety net hospital, and a regional medical center in a geographic region with firearm ownership rates that are higher than state averages. All three EDs had 24/7 coverage by behavioral health specialists. Study procedures occurred in the area where the patient was receiving clinical care to limit disruption to ED care and maintain safety precautions"

4b-i) Report if outcomes were (self-)assessed through online questionnaires

"At enrollment, participants completed an online baseline questionnaire and then viewed either (1) L2L on a web-enabled tablet computer or (2) the control, also on the tablet, consisting of general suicide prevention information without a focus on firearm or medication storage. All participants then completed a second questionnaire, including indicating their plan for firearm and medication storage and acceptability questions for the intervention group."

4b-ii) Report how institutional affiliations are displayed

No affiliations displayed

5) CONSORT: Describe the interventions for each group with sufficient details to allow replication, including how and when they were actually administered

5-i) Mention names, credential, affiliations of the developers, sponsors, and owners

NA - L2L is freely available

5-ii) Describe the history/development process

"The L2L web-based decision aid [19] was developed through an iterative process based on qualitative interviews with key stakeholders, including suicide prevention experts, members of the firearm community, survivors of suicide attempts, and loved ones of suicide victims, as described elsewhere [20]."

5-iii) Revisions and updating

"Based on initial feedback and low recruitment, and with IRB and DSMB approval, the study team modified L2L and the study protocol partway through the trial to also address medication safety in addition to firearms. Specifically, L2L incorporated a module on reducing access to medications and patients could be eligible if they reported medications at home (even without firearms); other eligibility criteria and study procedures were unchanged. "

5-iv) Quality assurance methods

NA

5-v) Ensure replicability by publishing the source code, and/or providing screenshots/screen-capture video, and/or providing flowcharts of the algorithms used

Website remains live

**5-vi) Digital preservation**		
Appendix includes screenshots		
**5-vii) Access**		
"At enrollment, participants completed an online baseline questionnaire and then viewed either (1) L2L on a web-enabled tablet computer" (tablet provided by study staff)		
**5-viii) Mode of delivery, features/functionalities/components of the intervention and comparator, and the theoretical framework**		
NA		
**5-ix) Describe use parameters**		
As needed, for adults with suicide risk (introduction)		
**5-x) Clarify the level of human involvement**		
"A self-administered decision aid "		
**5-xi) Report any prompts/reminders used**		
NA		
**5-xii) Describe any co-interventions (incl. training/support)**		
NA		
**6a) CONSORT: Completely defined pre-specified primary and secondary outcome measures, including how and when they were assessed**		
"Key measures for the intervention group assessed feasibility and acceptability. Feasibility was measured via minutes for the patient to complete L2L as measured by research staff, along with completion rate. Acceptability was measured using the Ottawa Acceptability Scale, a scale measuring comprehensibility (e.g., length, amount of information, balance in presentation, and overall suitability for decision making) [21]."<br><br>"Although the pilot trial was not powered to measure efficacy of L2L on decisions or behavior, for exploratory analysis we measured (1) decision conflict, a fundamental component of decision quality as a precursor to behavior change [22], and (2) behavior change itself. We hypothesized that patients with higher quality decisions (defined as lower decision conflict) after L2L would be more likely to change their home storage to reduce access to lethal means. Decision conflict was measured using the low-literacy version of the Decisional Conflict Scale (DCS), a 10-item scale with high reliability and test-retest correlation previously shown to discriminate between known groups who make or delay decisions [23]. The DCS scale is scored from 0 to 100, with lower scores indicating less decisional conflict. The baseline and follow-up questionnaires also recorded demographics, living situation, home firearms and medications, and suicide ideation or attempts as measured by the baseline and since-last-visit versions of the Columbia-Suicide Severity Rating Scale (C-SSRS) [24]. "		
**6a-i) Online questionnaires: describe if they were validated for online use and apply CHERRIES items to describe how the questionnaires were designed/deployed**		
**6a-ii) Describe whether and how "use" (including intensity of use/dosage) was defined/measured/monitored**		
"measured via minutes for the patient to complete L2L as measured by research staff"		
**6a-iii) Describe whether, how, and when qualitative feedback from participants was obtained**		
"Acceptability was measured using the Ottawa Acceptability Scale "		
**6b) CONSORT: Any changes to trial outcomes after the trial commenced, with reasons**		
"This pilot feasibility trial recruited participants from three large EDs in Colorado: a tertiary care academic center, an urban safety net hospital, and a regional medical center in a geographic region with firearm ownership rates that are higher than state averages. All three EDs had 24/7 coverage by behavioral health specialists. Study procedures occurred in the area where the patient was receiving clinical care to limit disruption to ED care and maintain safety precautions"		
**7a) CONSORT: How sample size was determined**		
**7a-i) Describe whether and how expected attrition was taken into account when calculating the sample size**		
pilot feasibility		
**7b) CONSORT: When applicable, explanation of any interim analyses and stopping guidelines**		
"Key measures for the intervention group assessed feasibility and acceptability. Feasibility was measured via minutes for the patient to complete L2L as measured by research staff, along with completion rate. Acceptability was measured using the Ottawa Acceptability Scale, a scale measuring comprehensibility (e.g., length, amount of information, balance in presentation, and overall suitability for decision making) [21]."<br><br>"Although the pilot trial was not powered to measure efficacy of L2L on decisions or behavior, for exploratory analysis we measured (1) decision conflict, a fundamental component of decision quality as a precursor to behavior change [22], and (2) behavior change itself. We hypothesized that patients with higher quality decisions (defined as lower decision conflict) after L2L would be more likely to change their home storage to reduce access to lethal means. Decision conflict was measured using the low-literacy version of the Decisional Conflict Scale (DCS), a 10-item scale with high reliability and test-retest correlation previously shown to discriminate between known groups who make or delay decisions [23]. The DCS scale is scored from 0 to 100, with lower scores indicating less decisional conflict. The baseline and follow-up questionnaires also recorded demographics, living situation, home firearms and medications, and suicide ideation or attempts as measured by the baseline and since-last-visit versions of the Columbia-Suicide Severity Rating Scale (C-SSRS) [24]. "		
**8a) CONSORT: Method used to generate the random allocation sequence**		
NA		
**8b) CONSORT: Type of randomisation; details of any restriction (such as blocking and block size)**		
block randomized		
**9) CONSORT: Mechanism used to implement the random allocation sequence (such as sequentially numbered containers), describing any steps taken to conceal the sequence until interventions were assigned**		
REDCap - "Randomization occurred after consent to minimize enrollment bias. "		
**10) CONSORT: Who generated the random allocation sequence, who enrolled participants, and who assigned participants to interventions**		
"Randomization occurred after consent to minimize enrollment bias." done by research staff via REDCap		
**11a) CONSORT: Blinding - If done, who was blinded after assignment to interventions (for example, participants, care providers, those assessing outcomes) and how**		
**11a-i) Specify who was blinded, and who wasn't**		
"Participants were blinded but research staff were not; to blind participants we used mild deception in the informed consent process such that patients knew the study was examining ways to enhance home safety of suicidal patients but did not know that L2L was the intervention of interest. Clinical staff were unaware of the treatment group."		
**11a-ii) Discuss e.g., whether participants knew which intervention was the "intervention of interest" and which one was the "comparator"**		
"Participants were blinded but research staff were not; to blind participants we used mild deception in the informed consent process such that patients knew the study was examining ways to enhance home safety of suicidal patients but did not know that L2L was the intervention of interest. Clinical staff were unaware of the treatment group."		
**11b) CONSORT: If relevant, description of the similarity of interventions**		
NA		
**12a) CONSORT: Statistical methods used to compare groups for primary and secondary outcomes**		
NA		
**12a-i) Imputation techniques to deal with attrition / missing values**		
NA		
**12b) CONSORT: Methods for additional analyses, such as subgroup analyses and adjusted analyses**		
"We used descriptive statistics for feasibility, acceptability, and exploratory analyses on DCS and behavior change. For continuous variables, differences in means between control and intervention groups were tested with two-sample t-tests with unequal variances. For categorical variables, we used frequencies with percentages, and differences between groups were tested with Fisher's exact test."		
RESULTS		
**13a) CONSORT: For each group, the numbers of participants who were randomly assigned, received intended treatment, and were analysed for the primary outcome**		
"Over 10 months 49 patients were enrolled, with 33 randomized to the L2L intervention group and 16 to the control group "		
**13b) CONSORT: For each group, losses and exclusions after randomisation, together with reasons**		
YES - diagram		
**13b-i) Attrition diagram**		
NA		
**14a) CONSORT: Dates defining the periods of recruitment and follow-up**		
"Two thirds (n=33; n=14 control and n=19 intervention) of participants completed telephone follow-up (Figure 1) at an average of 2.4 weeks (SD 2.2; range 1-9 weeks) after enrollment; "		
**14a-i) Indicate if critical "secular events" fell into the study period**		
NA		
**14b) CONSORT: Why the trial ended or was stopped (early)**		
NA		

**15) CONSORT: A table showing baseline demographic and clinical characteristics for each group**		
Yes - Table 1		
**15-i) Report demographics associated with digital divide issues**		
NA		
**16a) CONSORT: For each group, number of participants (denominator) included in each analysis and whether the analysis was by original assigned groups**		
**16-i) Report multiple “denominators” and provide definitions**		
YES - tables		
**16-ii) Primary analysis should be intent-to-treat**		
Yes		
**17a) CONSORT: For each primary and secondary outcome, results for each group, and the estimated effect size and its precision (such as 95% confidence interval)**		
"Feasibility and acceptability of the L2L intervention were excellent. All intervention group participants (n=33) completed L2L, with a median viewing time of 6 minutes (interquartile range: 4-10 minutes), and most (73%) wanted a print-out of the last page with final choices and recommendations. Most participants (93.9%) viewed L2L by themselves, without a family or friend present and without a provider. Figure 2 displays selected storage options; participants made 53 selections in L2L, in addition to “friends or family” (which was selected by default but could be unselected; 10 participants unselected it). Figure 3 shows responses to the Ottawa Acceptability Scale. Almost all participants reported that they would recommend the tool to a friend or family member in the same situation, that the options felt realistic, and that L2L was respectful of values about firearms. "		
**17a-i) Presentation of process outcomes such as metrics of use and intensity of use**		
"All intervention group participants (n=33) completed L2L, with a median viewing time of 6 minutes (interquartile range: 4-10 minutes"		
**17b) CONSORT: For binary outcomes, presentation of both absolute and relative effect sizes is recommended**		
YES - see tables		
**18) CONSORT: Results of any other analyses performed, including subgroup analyses and adjusted analyses, distinguishing pre-specified from exploratory**		
Yes		
**18-i) Subgroup analysis of comparing only users**		
Yes, acceptabiltiy results		
**19) CONSORT: All important harms or unintended effects in each group**		
NA no harms		
**19-i) Include privacy breaches, technical problems**		
NA none		
**19-ii) Include qualitative feedback from participants or observations from staff/researchers**		
Not available		
DISCUSSION		
**20) CONSORT: Trial limitations, addressing sources of potential bias, imprecision, multiplicity of analyses**		
**20-i) Typical limitations in ehealth trials**		
NA		
**21) CONSORT: Generalisability (external validity, applicability) of the trial findings**		
**21-i) Generalizability to other populations**		
Yes		
**21-ii) Discuss if there were elements in the RCT that would be different in a routine application setting**		
Yes		
**22) CONSORT: Interpretation consistent with results, balancing benefits and harms, and considering other relevant evidence**		
**22-i) Restate study questions and summarize the answers suggested by the data, starting with primary outcomes and process outcomes (use)**		
"In this pilot trial, acceptability of the “Lock to Live” decision aid was very high among adults with acute suicide risk. While the trial was not powered to identify an effect on subsequent home lethal means access, L2L appears feasible for clinical use in that it took a median of 6 minutes, and there were no major issues accessing the content via a tablet in the ED."		
**22-ii) Highlight unanswered new questions, suggest future research**		
" Questions remain, however, about how best to implement its routine use in clinical settings for appropriate patients"		
Other information		
**23) CONSORT: Registration number and name of trial registry**		
ClinicalTrials.gov NCT03478501		
**24) CONSORT: Where the full trial protocol can be accessed, if available**		
available on request		
**25) CONSORT: Sources of funding and other support (such as supply of drugs), role of funders**		
Yes		
**X26-i) Comment on ethics committee approval**		
"this study was approved through full board review of the Colorado Multiple Instructional Review Board"		
**x26-ii) Outline informed consent procedures**		
"The consent process included questions to determine cognitive capacity to consent. "		
**X26-iii) Safety and security procedures**		
"Study procedures occurred in the area where the patient was receiving clinical care to limit disruption to ED care and maintain safety precautions."		
**X27-i) State the relation of the study team towards the system being evaluated**		
Yes, none		
